# Ultrastructural analysis of mitochondrial morphology and in the human rhabdosphincter: Insights into urinary incontinence

**DOI:** 10.14814/phy2.70265

**Published:** 2025-03-02

**Authors:** Shinro Hata, Mayuka Shinohara, Hiromitsu Mimata, Toshitaka Shin

**Affiliations:** ^1^ Department of Urology Oita University Faculty of Medicine Yufu Oita Japan

**Keywords:** mitochondria, rhabdosphincter, urinary incontinence

## Abstract

Urinary incontinence increases with age, reducing the elderly quality of life. Understanding its mechanisms and developing treatments are urgent tasks. While healthy striated muscle maintains homeostasis through mitophagy, aging is thought to reduce autophagy activity. This study aimed to detect abnormal mitochondrial accumulation in the rhabdosphincter using transmission electron microscopy (TEM). We collected the rhabdosphincter samples from seven patients undergoing cystectomy and used the rectus abdominis as controls. Both tissues were examined with Hematoxylin and eosin (HE) staining and TEM. ImageJ software was used to measure the mitochondrial area, perimeter, and luminance. HE staining revealed that the rhabdosphincter had fewer muscle fibers and more stromal tissue than the rectus abdominis. TEM images showed more gaps in muscle bundles and signs of mitochondrial damage, vacuolation, and swelling in the rhabdosphincter. Quantitative analysis revealed a larger average mitochondrial area (0.21 μm^2^ vs. 0.063 μm^2^, *p* < 0.01), longer perimeter (1.83 μm vs. 0.94 μm, *p* < 0.01) and higher luminance (156.6 vs. 90.2, *p* < 0.01) than those of the rectus abdominis. The rhabdosphincter of elderly individuals showed significant mitochondrial morphological abnormalities, with increased swelling and vacuolation.

## 1 INTRODUCTION

Mitochondrial dysfunction is increasingly recognized as a critical factor in aging‐related disorders, particularly those affecting muscle function (López‐Otín et al., 2013). Mitochondria, the energy‐producing organelles in cells, have a vital role in maintaining cellular homeostasis through oxidative phosphorylation and mitophagy (Bratic & Larsson, 2013). Mitophagy removes damaged mitochondria and ensures proper function, but aging compromises mitochondrial quality‐control mechanisms, leading to structural and functional abnormalities (Palikaras et al., 2018). In particular, skeletal muscles, including the rhabdosphincter, exhibit significant mitochondrial dysfunction with aging, which may contribute to disorders such as sarcopenia and urinary incontinence (Romanello & Sandri, 2021). Studies have demonstrated that altered mitochondrial morphology, increased reactive oxygen species (ROS) production, and impaired ATP production are common in aged tissues and cause muscle weakening and functional decline (Chistiakov et al., 2014; Hyatt & Powers, 2021; Srivastava, 2017). Understanding the role of mitochondrial dysfunction in the rhabdosphincter is essential for elucidating the mechanisms of urinary incontinence in the elderly.

Despite significant advancements in understanding mitochondrial dysfunction, there are critical gaps in linking these changes to specific muscle pathologies, such as urinary incontinence in the elderly. Although mitochondrial dysfunction has been implicated in many aging‐related disorders progression, its precise role in rhabdosphincter degeneration is underexplored. Current research primarily focuses on general bladder dysfunction or smooth‐muscle impairment, with limited attention to the specific mitochondrial abnormalities in striated muscles, such as the rhabdosphincter (McDonnell et al., 2019). Additionally, studies linking mitochondrial dysfunction to urinary incontinence have highlighted the role of ROS production and mitochondrial DNA damage but have not extensively investigated the structural mitochondrial changes in the muscle tissues responsible for continence (Safir et al., 1998). These knowledge gaps emphasize the need for focused studies on mitochondrial morphology and function in urinary incontinence.

This study aimed to use transmission electron microscopy (TEM) to investigate the mitochondrial abnormalities in the rhabdosphincter of elderly individuals. By focusing on mitochondrial morphology, this research might fill in the current gap in our understanding of how structural changes in mitochondria relate to muscle dysfunction in the rhabdosphincter. Using TEM and quantitative analysis, we compared mitochondrial size, perimeter, and structural integrity to those of control tissues. The findings may provide novel insights into the role of mitochondrial dysfunction in age‐related urinary incontinence and offer potential pathways for therapeutic interventions targeting mitochondrial health in the elderly.

## 2 METHODS

### 2.1 Ethical approval

This study conformed to the standards set by the most recent version of the Declaration of Helsinki (except for registration in a database) and was approved by the Oita University Institutional Review Board (file no. 1947). All study participants provided oral and written informed consent.

### 2.2 Study population and sample collection

The study included seven patients (5 males and two females) with a median age of 76.5 years (68–91 years). All patients were undergoing radical cystectomy for bladder cancer between March 2022 and May 2023 and met the inclusion criteria of appropriate age and absence of neuromuscular diseases. Samples of the rhabdosphincter and rectus abdominis (the control) were collected during surgery. Both tissue samples were immediately fixed in 2.5% glutaraldehyde for TEM analysis. Table 1 summarizes the demographic and clinical characteristics of the study participants, including age, sex, body mass index (BMI), exercise habits, International Prostate Symptom Score (IPSS, range, 0–35, with higher scores indicating severe lower urinary tract symptoms), and Overactive Bladder Symptom Score (OABSS, range, 0–15, with higher scores indicating severe urinary incontinence).

**TABLE 1:** Demographic and clinical characteristics of the study participants.

No.	Age	Sex	BMI, kg/m^2^	Excersise habits	IPSS	OABSS
1	70	M	21.3	None	7	8
2	69	M	15.7	None	14	3
3	68	M	21.8	Daily walking	^a^	^a^
4	91	F	20.2	None	24	4
5	84	M	23.4	None	3	1
6	87	F	16.9	None	13	8
7	81	M	23.4	Dairy farming work	5	6

Abbreviations: BMI; body mass index, IPSS; International Prostate Symptom Score, OABSS; Overactive Bladder Symptom Score.

^a^ Data not available due to anuria in a dialysis patient.

### 2.3 Tissue preparation and staining

After tissue collection, the rhabdosphincter and rectus abdominis samples were fixed in 2.5% glutaraldehyde at 4°C for 24 h. The tissues were then rinsed in phosphate buffer (0.1 M, pH 7.4) and then fixed in 1% osmium tetroxide for 1 h. After dehydration through an ascending ethanol series, the tissues were embedded in epoxy resin. Semi‐thin sections (1 μm) were stained with toluidine blue for light microscopy, and ultrathin sections (70 nm) were stained with uranyl acetate and lead citrate for TEM analysis.

### 2.4 TEM analysis

A HITACHI H‐7650 TEM was used to examine ultra‐thin sections of the samples. Images were captured at 120 kV, and representative micrographs were taken at 5000× and 20,000× magnification to evaluate the mitochondrial structure. The analysis focused on identifying the key features of mitochondrial morphology, such as swelling, vacuolation, and fragmentation. All TEM images were saved as high‐resolution TIFF files for subsequent quantitative analysis.

### 2.5 Mitochondrial morphology quantification

To quantify the mitochondrial morphology, ImageJ software was used to measure the mitochondrial area, perimeter, and luminance in both samples. Three random tissue sections were selected for each participant, and three mitochondria were measured from each section for the rhabdosphincter and rectus abdominis samples. This resulted in nine mitochondrial measurements per patient per tissue type (3 mitochondria × 3 sections), totaling 63 measurements per group (9 measurements × 7 patients). To ensure unbiased selection, tissue sections and mitochondria were selected and measured by researchers blinded to sample information. To account for the hierarchical structure of the data (mitochondria nested within sections, and sections nested within patients) and the non‐independence of measurements, we used a mixed‐effects model with patient ID and section ID as random effects in our statistical analysis. The average mitochondrial area (in μm^2^), perimeter (in μm) and luminance (in grayscale units) were calculated and compared between the two tissue types. Statistical comparisons between the groups were performed to determine the significant differences in mitochondrial size and structure.

### 2.6 Statistical analysis

All data are presented as the mean. To account for the hierarchical structure of the data (mitochondria nested within sections, and sections nested within patients), a linear mixed‐effects model was used with patient ID and section ID as random effects. The differences in mitochondrial morphology parameters between the rhabdosphincter and rectus abdominis muscle tissues were analyzed. Values of *p* < 0.05 were accepted as indicating statistical significance. All statistical analyses were performed using R version 4.2.0 (R Foundation for Statistical Computing, Vienna, Austria) with the ‘lme4’ package for mixed‐effects modeling.

## 3 RESULTS

**FIGURE 1:**
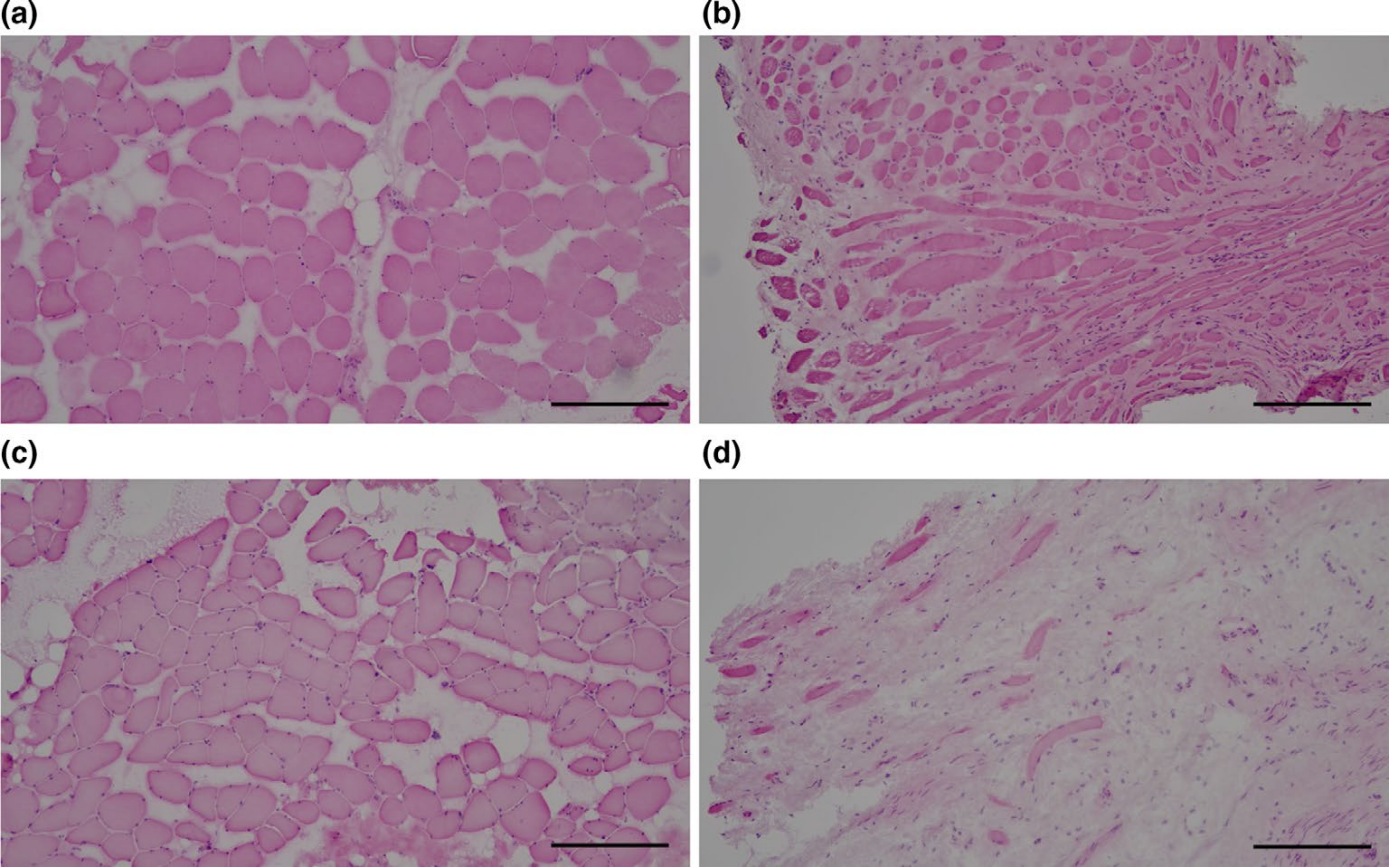
Histological comparison of the rhabdosphincter and rectus abdominis muscle architecture. Hematoxylin and eosin (HE) staining of skeletal muscle sections from the rectus abdominis (a, c) and rhabdosphincter tissues (b, d). The rhabdosphincter tissue shows reduced muscle fiber density and increased stromal tissue content relative to those in the rectus abdominis. Images (a) and (b) were obtained from the same individual as were (c) and (d). Scale bar = 100 μm.

**FIGURE 2:**
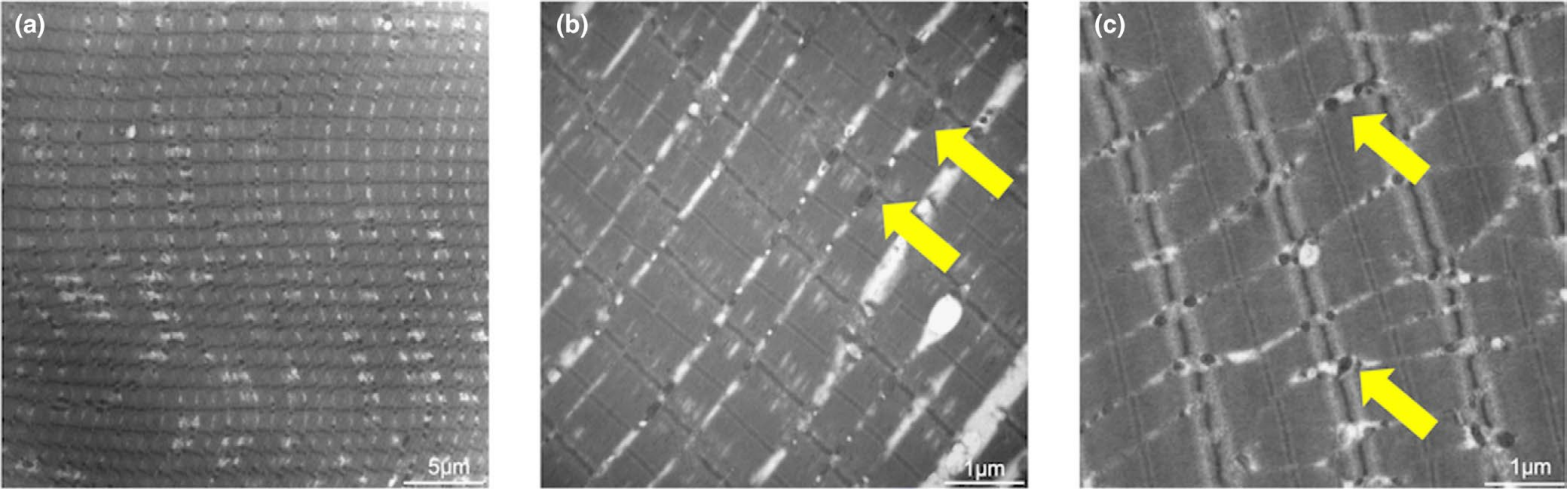
TEM of the rectus abdominis muscle. TEM images showing the rectus abdominis muscle's ultrastructural features with normal mitochondrial distribution and muscle bundle organization. Yellow arrows indicate mitochondria.

**FIGURE 3:**
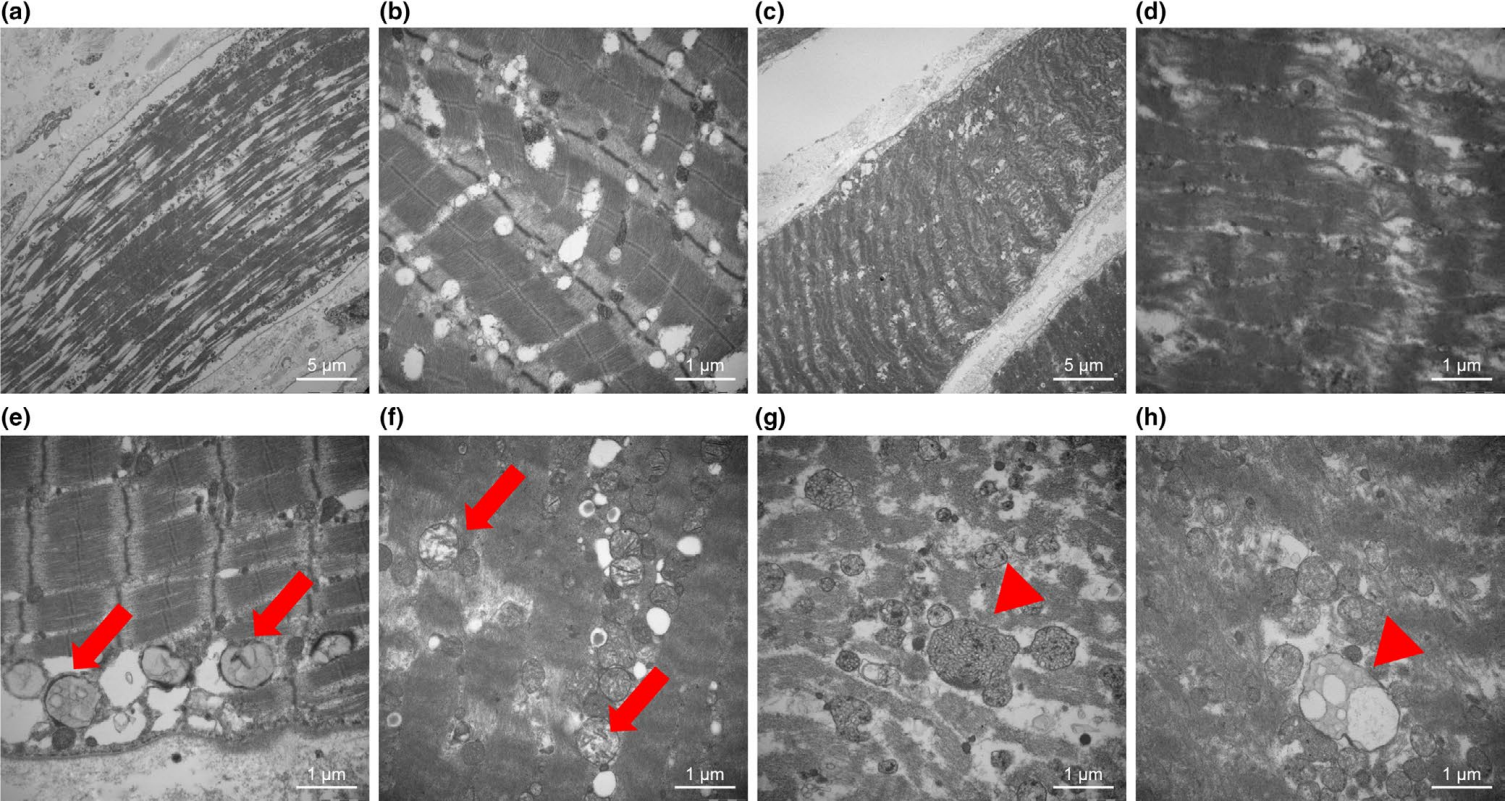
Ultrastructural alterations in the rhabdosphincter revealed by TEM. TEM images showing pathological changes in the rhabdosphincter. (a, b) Increased gaps between muscle bundles. (c, d) Reduced mitochondrial density relative to that in the rectus abdominis. (e, f) Mitochondrial vacuolation (red arrows). (g, h) Mitochondrial swelling (red arrowheads). These alterations suggest abnormal mitochondrial accumulation and structural disorganization.

HE staining revealed significant structural differences between the rhabdosphincter and rectus abdominis. The rhabdosphincter exhibited fewer muscle fibers and increased stromal tissue, suggesting a predisposition to functional decline (Figure 1). TEM images further supported these findings, showing more gaps between muscle bundles and clear signs of mitochondrial damage, vacuolation, and swelling in the rhabdosphincter, indicating abnormal mitochondrial accumulation (Figures 2 and 3).

**FIGURE 4:**
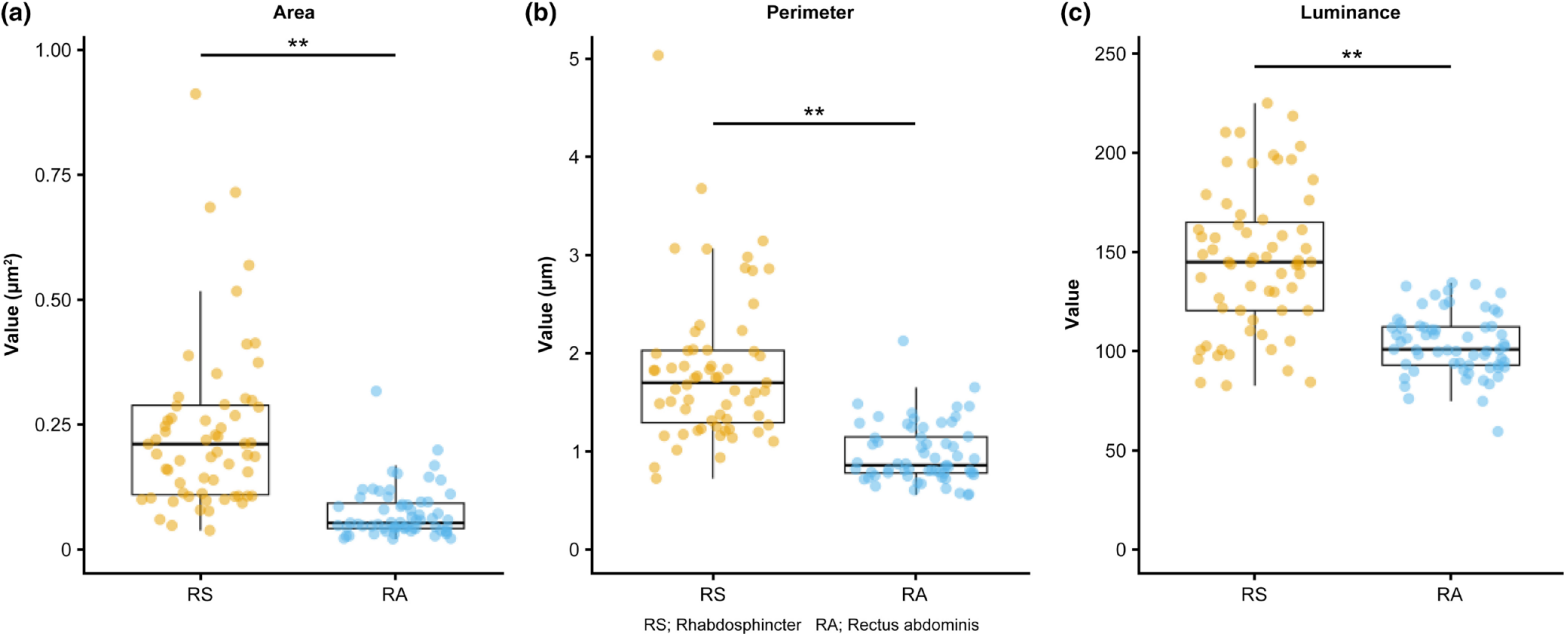
Quantitative analysis of the mitochondrial morphology. Comparison of mitochondrial parameters between the rhabdosphincter and rectus abdominis. Box plots with individual data points showing the (a) mitochondrial area (μm^2^), (b) perimeter (μm), and (c) luminance values. Each datapoint represents a single mitochondrial measurement, with nine measurements per patient (three mitochondria measured from each of three randomly selected tissue sections). The rhabdosphincter showed a significantly larger mean mitochondrial area (0.21 μm^2^ vs. 0.063 μm^2^), longer mean perimeter (1.83 μm vs. 0.94 μm) and higher mean luminance values (156.6 vs. 90.2) than those in the rectus abdominis. Box plots indicate the mean value (horizontal line), 95% confidence intervals (boxes), and whiskers (minimum to maximum). **p < 0.01.

Quantitative analysis of mitochondrial morphology also showed significant differences between the rhabdosphincter and rectus abdominis. The average mitochondrial area was significantly larger (0.21 μm^2^ vs. 0.063 μm^2^, *p* < 0.01), and the perimeter was longer (1.83 μm vs. 0.94 μm, *p* < 0.01) in the rhabdosphincter than in the rectus abdominis (Figure 4). Additionally, the luminance of the mitochondria, indicating mitochondrial density and potential oxidative damage, was significantly higher in the rhabdosphincter (156.6 vs. 90.2, *p* < 0.01). These findings indicate substantial mitochondrial abnormalities in the rhabdosphincter, which may contribute to functional decline and urinary incontinence in elderly individuals.

## 4 DISCUSSION

This study identified significant mitochondrial abnormalities in the rhabdosphincter of elderly individuals, which may contribute to urinary incontinence. Through histological and TEM analyses, fewer muscle fibers, more stromal tissue, and increased mitochondrial swelling, vacuolation, and signs of mitochondrial damage were observed in the rhabdosphincter than in the control rectus abdominis muscle. Quantitative analysis further demonstrated a larger mitochondrial area, a longer perimeter, and a higher luminance in the rhabdosphincter than in the control. These findings suggest that age‐related mitochondrial dysfunction has a critical role in the decrease in muscle function in the rhabdosphincter, leading to urinary incontinence in elderly patients.

The first major finding of this study was the observation of significant enlargement and structural abnormalities of the mitochondria in the rhabdosphincter relative to those of the rectus abdominis. The increased mitochondrial area and perimeter, along with signs of vacuolation and swelling, suggest disrupted mitochondrial dynamics, potentially driven by an imbalance in the mitochondrial fission and fusion processes. Similar mitochondrial abnormalities have previously been found to be associated with impaired muscle function in aging‐related conditions, such as sarcopenia, in which altered mitochondrial morphology contributes to muscle weakness and degeneration (Affourtit & Carré, 2024; Bellanti et al., 2021; Marzetti et al., 2013). The mitochondrial swelling and vacuolation observed in the current study are consistent with previous findings indicating that oxidative stress and impaired mitophagy may be crucial factors contributing to mitochondrial dysfunction in aging tissues (McDonnell et al., 2019). These mitochondrial changes probably reduce energy production and contribute to muscle degeneration in the rhabdosphincter, ultimately impairing its function.

The second major study finding was the significantly higher mitochondrial luminance in the rhabdosphincter than in the rectus abdominis, suggesting potential oxidative damage or alterations in mitochondrial density. Increased mitochondrial luminance may reflect a higher concentration of ROS and oxidative stress, both of which are known to accumulate with age and impair mitochondrial function. Excessive ROS can promote the opening of mitochondrial permeability transition pores (mPTP) in the inner mitochondrial membrane, leading to matrix swelling, cristae disruption, and vacuolisation, which are observed as increased mitochondrial luminance in TEM images (Szabo et al., 2010; Zorov et al., 2000). Oxidative stress can cause mitochondrial DNA mutations and damage the mitochondrial membrane, further disrupting mitochondrial dynamics and energy production. Previous studies have linked elevated ROS levels to muscle weakness and degeneration in aging tissues (Chistiakov et al., 2014). In the rhabdosphincter, this oxidative damage could contribute to muscle atrophy and impaired contractility, ultimately leading to urinary incontinence in elderly individuals.

The accumulation of abnormal mitochondria in the rhabdosphincter observed in this study may be linked to impaired mitophagy, which is a crucial process for mitochondrial quality control. Mitophagy is responsible for the selective degradation of damaged mitochondria, preventing the buildup of dysfunctional organelles. In aging tissues, including skeletal muscles, mitophagy is often disrupted, leading to the accumulation of defective mitochondria and contributing to age‐related muscle dysfunction (Chen et al., 2020; Leduc‐Gaudet et al., 2021). Reduced mitophagy has been linked to sarcopenia progression, which is characterized by loss of muscle mass and strength with aging (Affourtit & Carré, 2024; Bellanti et al., 2021; Marzetti et al., 2013). Impaired mitophagy in the rhabdosphincter could lead to the buildup of damaged mitochondria that reportedly contributed to previously observed morphological abnormalities and muscle dysfunction (De Gaetano et al., 2021). Future studies targeting mitophagy pathways may offer valuable understanding and knowledge that could guide the development of therapeutic strategies designed to reestablish mitochondrial homeostasis in age‐related urinary incontinence.

While these findings provide important insights into the potential role of mitochondrial dysfunction in age‐related urinary incontinence, we acknowledge certain limitations. One such limitation was the relatively small sample size, which should be considered when evaluating our findings and highlights the need for further investigation into the molecular and cellular mechanisms connecting mitophagy regulation to rhabdosphincter function. This small sample size also prevented us from performing a more detailed analysis of the potential influence of factors such as BMI and exercise habits on mitochondrial morphology. Future research should aim to identify specific factors controlling mitophagy in the rhabdosphincter and their effects on muscle integrity and function, ideally with larger cohorts and more comprehensive assessments of body composition and physical activity levels.

In addition to the sample size, another limitation lies in the inherent differences between the rhabdosphincter and the rectus abdominis muscles used as a control. The rhabdosphincter predominantly comprises slow‐twitch fibers, known to contain larger and more numerous mitochondria than the fast‐twitch fibers found in the rectus abdominis (Kayar et al., 1988). While we used the rectus abdominis as a readily available control from the same individuals, it is important to acknowledge that inherent differences in fiber type composition could contribute to the observed differences in mitochondrial morphology. Ideally, future studies would compare the rhabdosphincter to younger rhabdosphincter samples. However, obtaining such samples presents significant ethical and logistical challenges due to the invasive nature of the biopsy and potential risks to urinary continence function in healthy volunteers. It would be ethically unjustifiable to subject healthy volunteers to this risk solely for research purposes.

Furthermore, while this study compared mitochondrial morphology between the rhabdosphincter and rectus abdominis, it is important to note that the rhabdosphincter may have a unique developmental origin, potentially arising from the transdifferentiation of smooth muscle (Borirakchanyavat et al., 1997). This difference in origin could potentially contribute to the observed mitochondrial abnormalities. However, age‐related changes are also a likely contributing factor, as mitochondrial function is known to decline with age in various muscle types (Short et al., 2005). Due to these factors, we could not definitively separate the effects of developmental origin from those of aging on mitochondrial morphology in this study.

It is also important to acknowledge that the rectus abdominis samples, while serving as a control, were also obtained from elderly individuals undergoing surgery for bladder cancer. Therefore, we cannot definitively rule out the possibility that some degree of mitochondrial alteration may also be present in the control tissue due to age or underlying disease processes. Given these limitations, our findings suggest a potential association between altered mitochondrial morphology in the rhabdosphincter and age‐related urinary incontinence; further research is needed to confirm this link and to emphasize the precise mechanisms involved. This study provides a valuable foundation for future investigations, highlighting the need for careful consideration of muscle type, developmental origin, and age‐related changes when studying mitochondrial function in the context of urinary incontinence.

## AUTHOR CONTRIBUTIONS

Shinro Hata: First draft, data analysis, data interpretation, table preparation, and revisions. Mayuka Shinohara: Data collection, data interpretation, and revisions. Hiromitsu Mimata: Conception, data collection, data interpretation, revisions, and supervision. Toshitaka Shin: First draft writing, data analysis, data interpretation, revisions, and supervision.

## FUNDING INFORMATION

This research was supported by JSPS KAKENHI Grant Number 24K19670.

## CONFLICT OF INTEREST STATEMENT

The authors declare that there are no conflicts of interest.

## ETHICS STATEMENT

This study confirmed to the standards set by the most recent version of the Declaration of Helsinki (except for registration in a database) and was approved by the Oita University Institutional Review Board (file no. 1947).

## Data Availability

The data underlying our findings can be shared upon reasonable request directed to the corresponding author.
